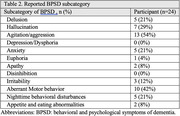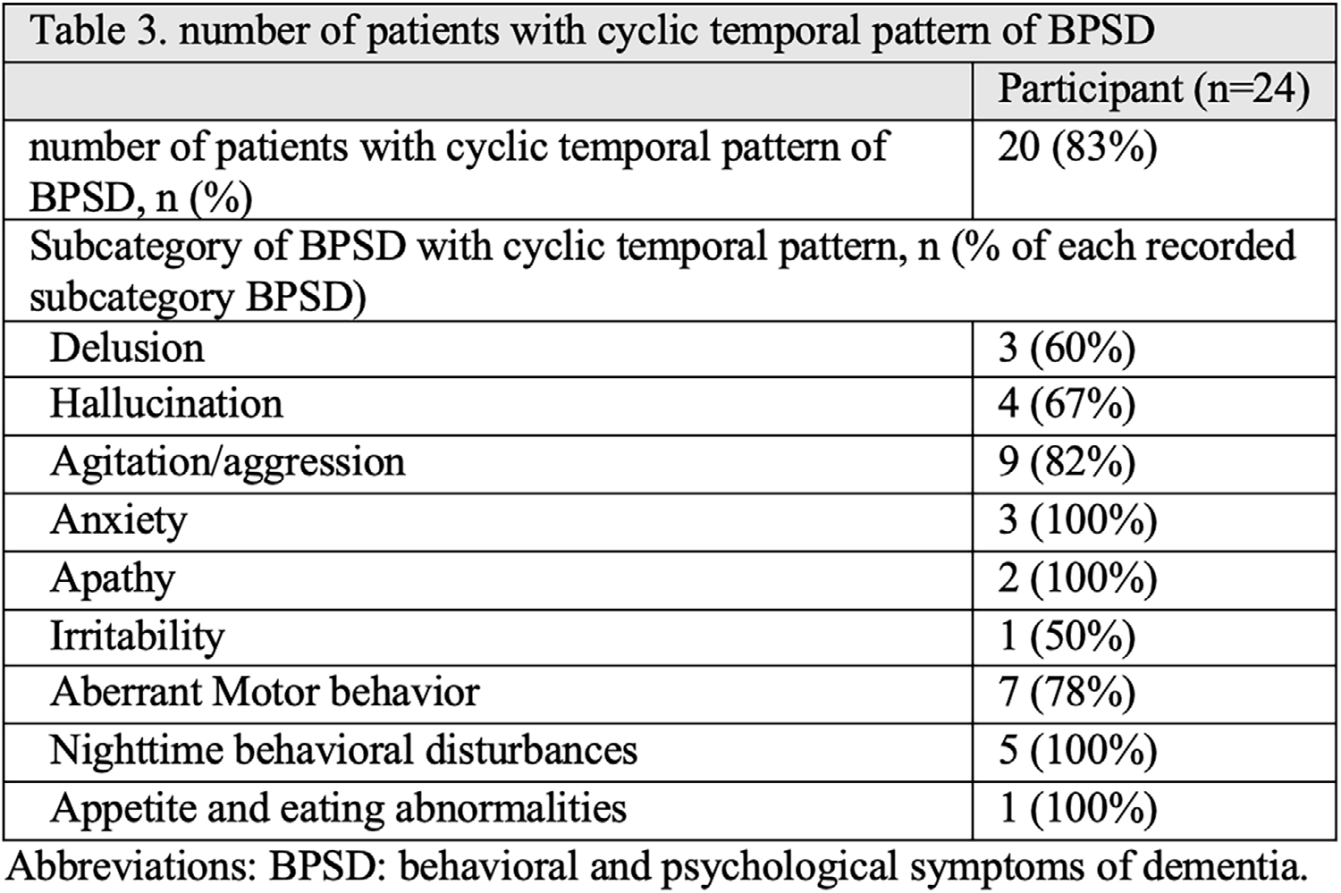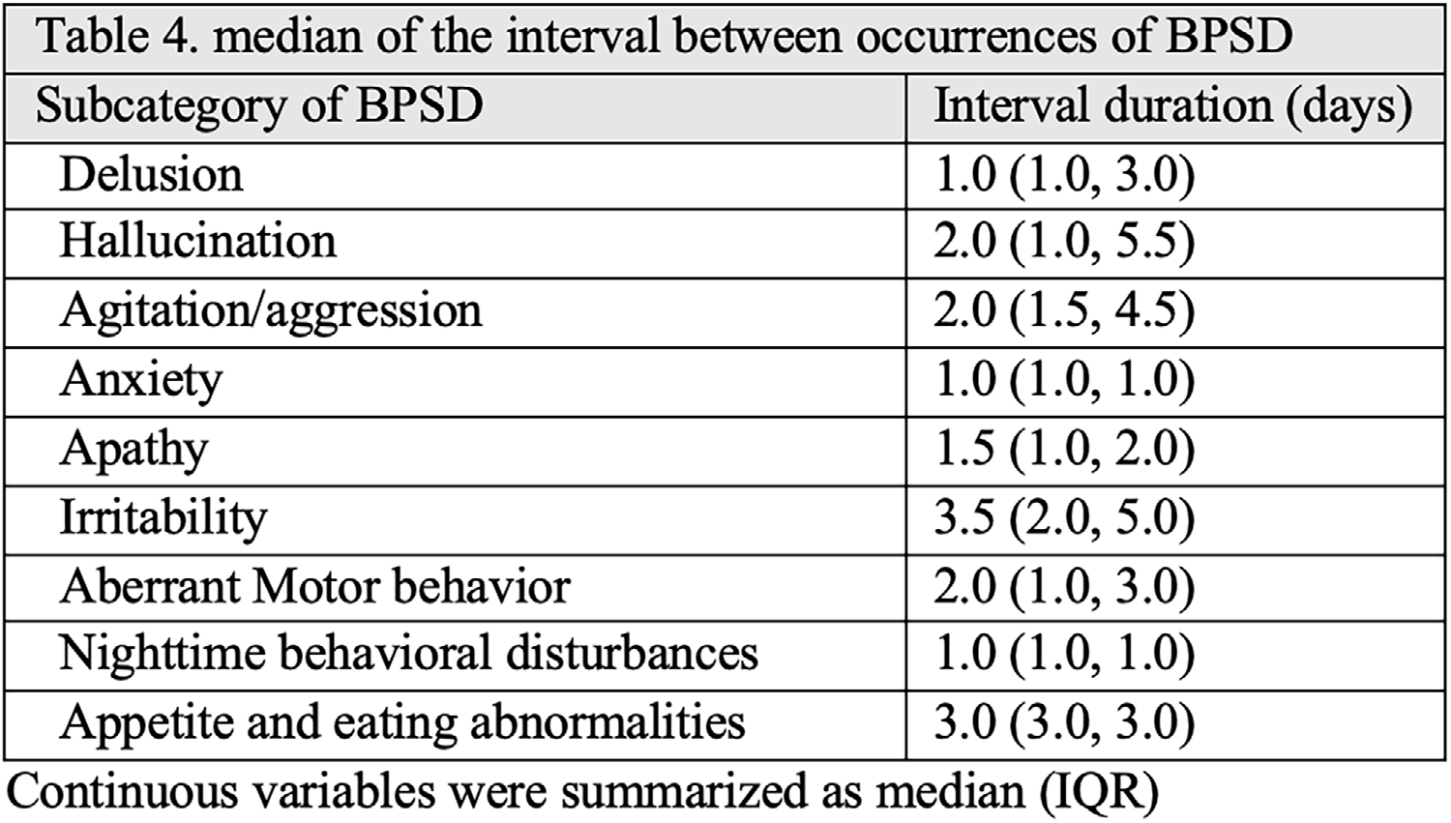# Prevalence of people living with dementia with cyclic temporal pattern of behavioral and psychological symptoms of dementia in a tertiary memory clinic: A descriptive diary‐based study

**DOI:** 10.1002/alz.085858

**Published:** 2025-01-03

**Authors:** Tara Rak‐Areekul, Yuttachai Likijaroen, Akarin Hiransuthikul, Kittithatch Booncharoen, Yuthachai Sarutikriangkri

**Affiliations:** ^1^ Faculty of Medicine, Chulalongkorn university, Bangkok, Bangkok Thailand; ^2^ Chulalongkorn university, Bangkok, Bangkok Thailand; ^3^ Faculty of Medicine, Chulalongkorn University, Bangkok Thailand; ^4^ Memory Clinic, Division of Neurology, King Chulalongkorn Memorial Hospital, Bangkok Thailand; ^5^ Department of Preventive and Social Medicine, Faculty of Medicine, Chulalongkorn University, Bangkok Thailand; ^6^ Memory Clinic, King Chulalongkorn Memorial Hospital, Bangkok Thailand; ^7^ Neurocognitive Unit, Division of Neurology, Faculty of Medicine, Chulalongkorn University, Bangkok Thailand

## Abstract

**Background:**

Behavioral and psychological symptoms of dementia (BPSD) profoundly impact individuals with dementia and their caregivers. Despite existing pharmacological interventions, symptom control remains challenging, often prompting polypharmacy with potential risks. Notably, in clinical practice, caregivers frequently report recurring patterns in patients' BPSD but lack empirical studies supporting these observations. The primary objective of our research is to explore the prevalence of predictable, cyclic BPSD patterns to enhance symptom control and minimize risks associated with excessive medication use.

**Method:**

Implementing a diary‐based approach, we recruited 24 dementia patients attending a memory clinic. Participants documented patients’ BPSD occurrences using hourly recordings across a 28‐day period. We categorized and quantified BPSD subcategories based on the Neuropsychiatric Inventory Questionnaire (NPI‐Q) framework. Cyclic patterns were defined by the recurrence of BPSD in at least 3 episodes, showing consistent intervals within a permissible range exceeding 50% of all episodes. This range allowed a deviation of up to 50% from the median interval.

**Result:**

Strikingly, 83% (n = 20) of participants exhibited cyclic BPSD patterns. Among the various BPSD subcategories, anxiety, apathy, nighttime behavioral disturbances, and appetite/eating abnormalities emerged as the most prominent features within these predictable fluctuations. Median intervals varied among subcategories, with irritability exhibiting the longest duration (3.5 days) and anxiety, delusion, and nighttime disturbances displaying the shortest (1.0 day), supporting the biopsychosocial model of BPSD pathogenesis.

**Conclusion:**

Cyclic BPSD patterns are surprisingly common, suggesting potential for personalized medication schedules based on predicted symptom peaks. Further research with larger populations is needed to validate these findings and develop tailored interventions for improved dementia care.